# Supplementary material for: Factors Influencing the Emergence of Dependency Length Minimization in Neural Agent Simulations
Source: Cogn Sci. 2026 Oct 7;50(10):e70269. doi: 10.1111/cogs.70269 (PMC13643502; doi:10.1111/cogs.70269)
Supplement: Supplementary file 1 [file COGS-50-e70269-s001.pdf]

□

APPENDIX

A DATA GENERATION DETAILS

To generate the skewed version of each language, we use the following discrete distributions which roughly correspond to ten equally spaced sample points from the Zipf distribution by setting the number of elements N to 10 and the exponent parameter s to 1.5: P_{agent} = [0.501, 0.177, 0.096, 0.063, 0.045, 0.034, 0.027, 0.022, 0.019, 0.016] for the probabilities of ten randomly shuffled animate nouns being agent given each verb, and the same distribution for the probabilities of ten randomly shuffled animate nouns being patient given each verb. By contrast, the uniform versions assign an equal probability to all animate nouns for being agents or patients with each verb.

In order to make a controlled setting and analyze the impact of skewed distribution on the development of DLM preferences in neural learners, we simplify the artificial language by assuming that all meaning tokens, except for agent and patient, have equal probability.

For each action, the meanings for both SM and OM spaces are generated by first exhaustively combining all available adpositions, adjectives, and inanimate nouns, resulting in $4 * 3 * 3 = 36$ unique combinations. Each of these combinations is duplicated to augment the data and facilitate the training process. Each combination is further expanded by concatenating it with an agent. The agents are sampled based on their respective conditional word distributions with the action, resulting in $72*0.501+72*0.177+72*0.096+72*0.063+72*0.045+72*0.034+72*0.027+72*0.022+72*0.019+72*0.016 \approx 67$ combinations. This new combination is duplicated and expanded by adding the meaning category of the patient. This is done by sampling patients based on their conditional probability ratios with the action, resulting in $134 * 0.501 + 134 * 0.177 + 134 * 0.096 + 134 * 0.063 + 134 * 0.045 + 134 * 0.034 + 134 * *0.027 + 134 * 0.022 + 134 * 0.019 + 134 * 0.016 \approx 129$ combinations. A total of $129 * 10(actions) * 2(SM/OM) = 2580$ meanings are generated. Similar to human experiments, we assume that the agent and patient are never the same within a single meaning. Therefore, certain meanings are filtered out, and repeated meanings are excluded as well. We sample half of the SM meanings and then generate short dependency utterances, and then generate long dependency utterances for the other half of the SM meanings. Then we repeat the same steps for OM meanings.

Generating data using this simple method can guarantee that items in categories other than Action, agent, and patient occur with equal probability and that the conditional probabilities of action, agent, and patient obey the assigned discrete skewed distribution. This process results in a comprehensive set of meaning structures that are representative of the dependency relationships in real languages.

For the uniform version, we generate all possible combinations of verbs, subjects, objects, and their modifiers. Then randomly sample an equal number of meanings. Data distributions are shown in Figure A1.

B INDIVIDUAL PRODUCTION PATTERNS UNDER THE NOISY CONDITION IN EXPERIMENT 1

This appendix includes the production patterns of 20 speaking agents (random seeds) after learning or communication for the verb-initial *skewed mixed* language, word dropout 0.1 (Figure B2). Around half regularize towards SO word order and another half regularize towards OS word order. The production patterns for other settings (verb-final, uniform languages, with dropout) are all similar in terms of regularization strategies and are therefore not shown here.

This appendix includes the production patterns of 40 speaking agents (random seeds) during communication learning for the verb-initial *skewed mixed* language with only SM meaning space.

C PER-CATEGORY LISTENING ACCURACY IN THE VERB-INITIAL LANGUAGE

This appendix shows the listening accuracy computed separately for each individual meaning item and the average listening accuracy in the verb-initial language as a function of the training epoch (Figure C3).

D LEARNING AND COMMUNICATION ACCURACIES IN THE VERB-FINAL LANGUAGE

As shown in Figure D4, mostly similar results were found for verb-initial and verb-final languages regarding learning and communication accuracies.

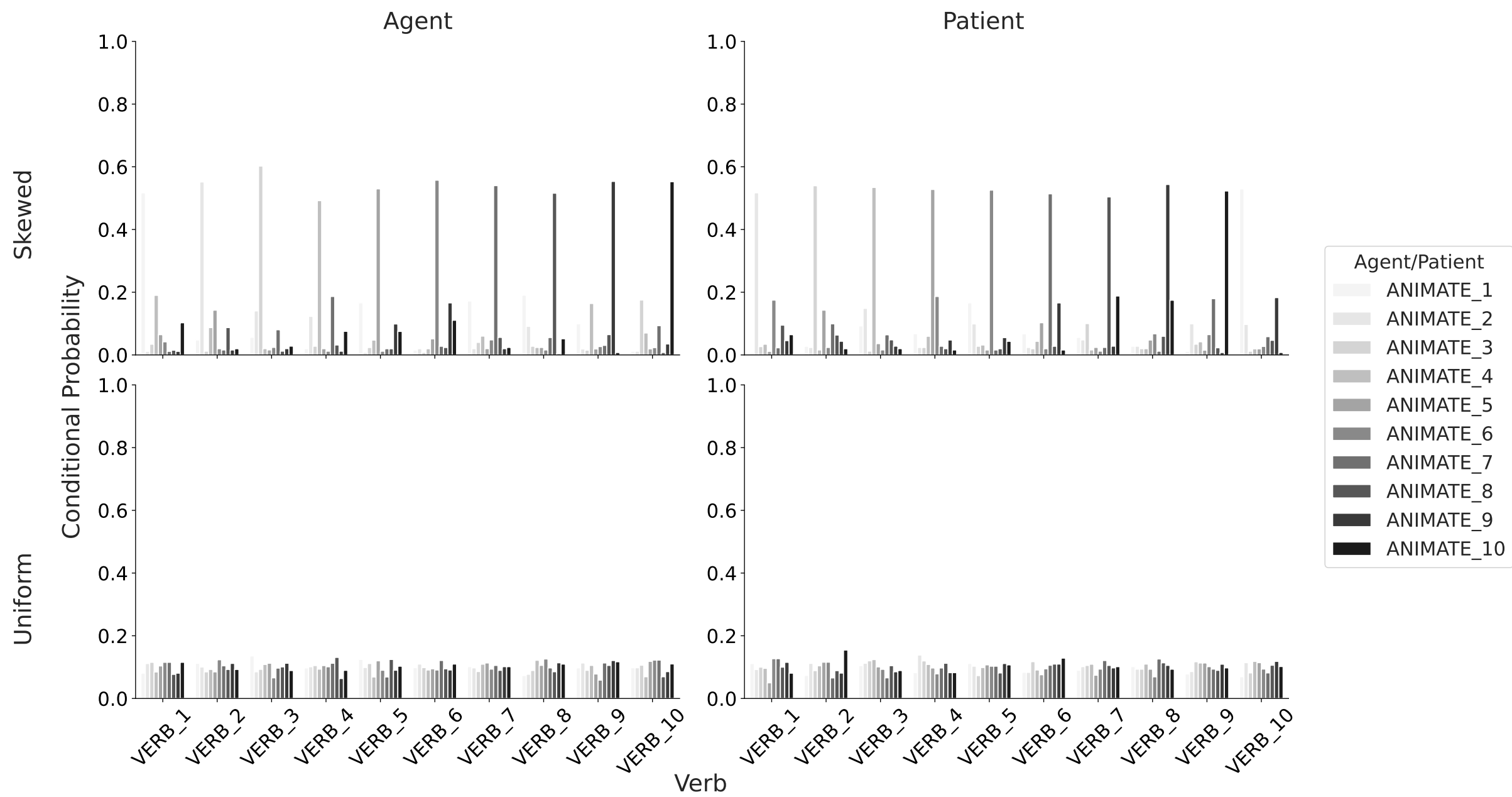

FIGURE A1 Conditional probability of Agent (left) and Patient (right) given Actions for skewed (top) and uniform (bottom) languages.

E ERROR ANALYSES

To better understand agents' production behavior, we conducted error analyses of their production during communication (After 60 RL epochs). Figure E5 illustrates the evolution of error percentages for all meaning items across epochs in the two languages. Error rates were calculated separately for utterances with short and long dependencies under three experimental conditions: *Baseline*, *Lower speaker capacity*, and *Noise*. The error percentage for meaning item categories was calculated by dividing the number of prediction errors by the total number of generated utterances.

For the verb-initial language, in the *Baseline* condition (top row of Figure E5), agents gradually achieved lower error rates for short dependencies (VOS) while maintaining higher rates for long dependencies (VSO). At the beginning of communication, agents produced an equal mix of SO and OS utterances, resulting in slightly higher error rates for meaning items, especially for agent and patient. Over time, the proportion of short DL utterances surpassed that of long DL utterances. In line with our expectations, the overall error rates for agent and patient decreased. For the verb-final language, in the *Baseline* condition (top row of Figure E5), agents gradually achieved lower error rates for long dependencies (OSV) while maintaining higher rates for short dependencies (SOV)[†].

The overall pattern suggested that a large portion of errors stemmed from role ambiguity, specifically misidentification of the agent or patient. For short-dependency utterances in the verb-initial language, while the initial error rate for agent or patient misidentification was relatively high, it declined noticeably over time, eventually reaching its lowest point compared to long-dependency utterances. The reversed pattern was found for the verb-final language.

In the lower speaker capacity condition (middle row) and the *Noise* condition (bottom row), error rates were decreasing, but less strongly than in the *Baseline* condition, with an overall higher error percentage. This pattern indicated that limited speaker capacity and noise hindered agents' ability to recover meanings. Despite this, the trends in agent and patient misidentification remained similar to the *Baseline* condition.

[†] In the verb-final language, agents with lower speaker capacity only produced long-dependency utterances as of epoch 30, causing the error percentage in Figure **??** to drop to zero.

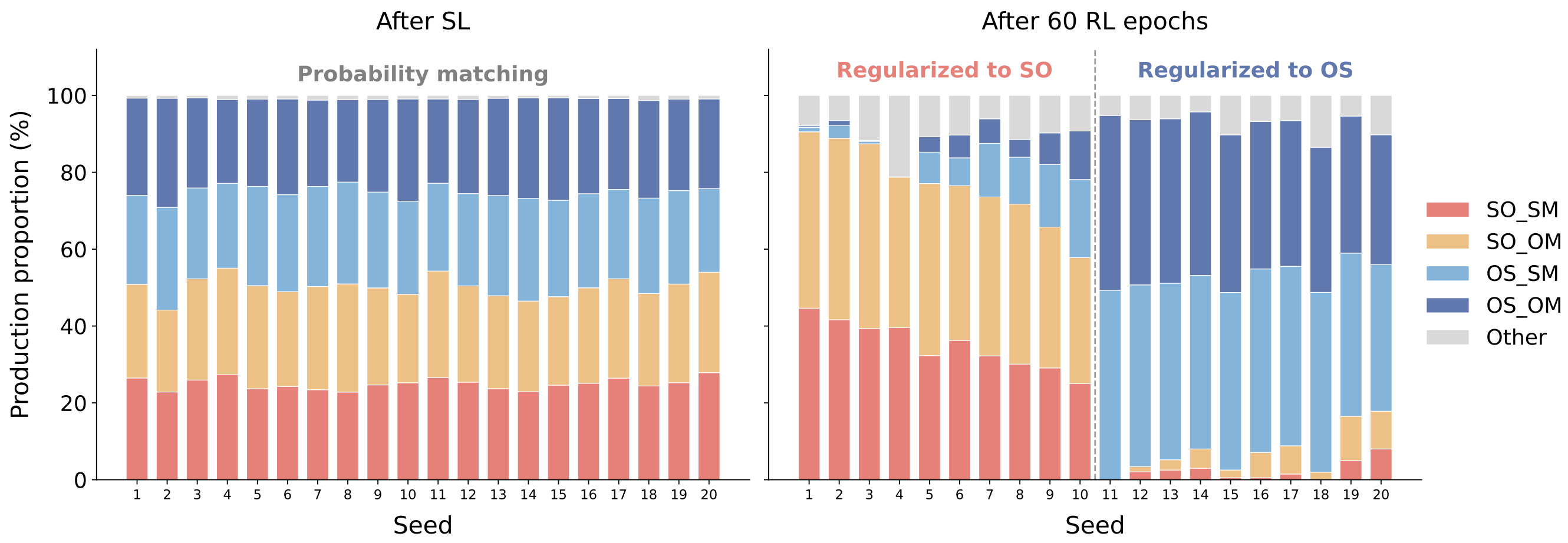

FIGURE B2 Seed-level production patterns before and after communication for the **verb-initial** *skewed mixed* language with the **full meaning space** and **dropout rate 0.1** (Noise condition). Each vertical bar represents one random seed and sums to 100% of that seed's productions. Colors encode both word order and meaning type: SO_SM, SO_OM, OS_SM, and OS_OM denote productions using SO or OS order for subject-modified (SM) or object-modified (OM) meanings, respectively; grey denotes other or incorrect productions. The left panel shows productions after SL, where agents largely preserve the mixed input distribution, consistent with probability matching. The right panel shows productions after 60 communication epochs, where seeds are sorted by their dominant word order and individual agents regularize toward either SO or OS. This pattern shows that, under dropout, communication still induces word-order regularization at the seed level, even though the aggregate proportion of local-dependency utterances can remain close to the 50% input baseline.

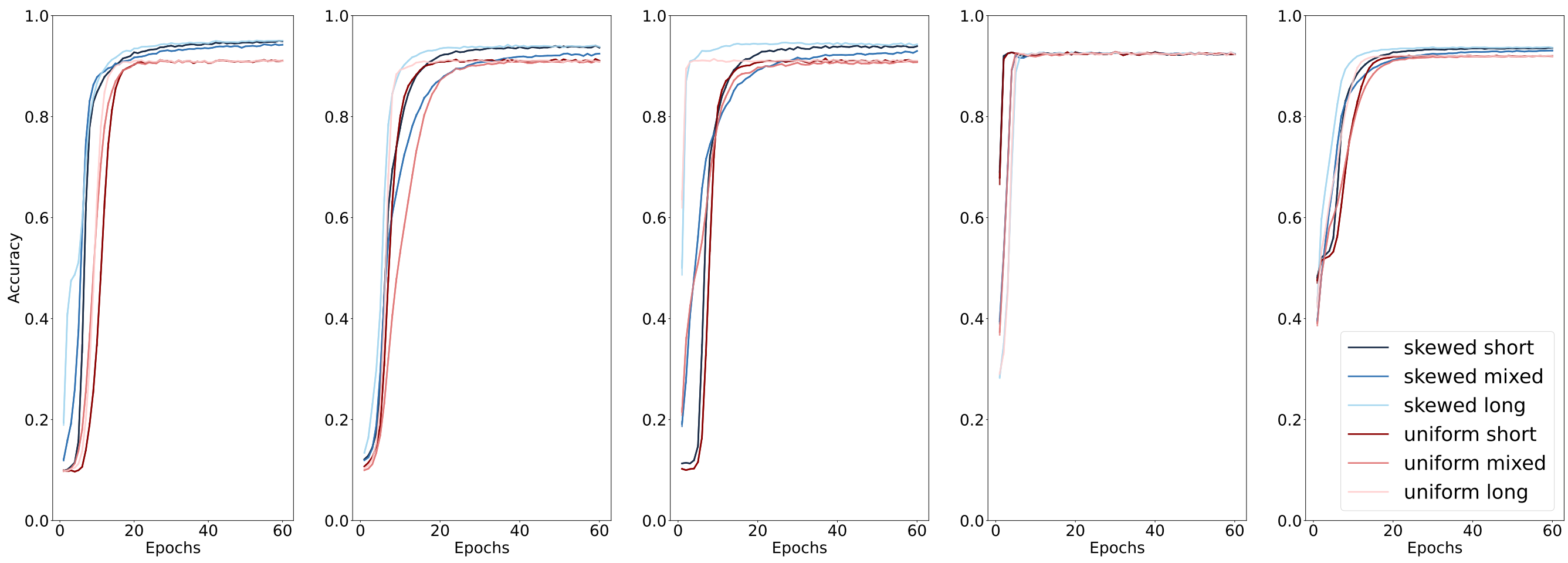

(a) Verb (b) Subject (c) Object (d) Modifier 1 (e) Avg Acc

FIGURE C3 Listening accuracy computed separately for each individual meaning item in the verb-initial language, as a function of the training epoch. We see similar results for $\{\mu_a^1, \mu_a^2, \mu_a^3\}$. Therefore, only μ_a^1 is plotted here for illustration.

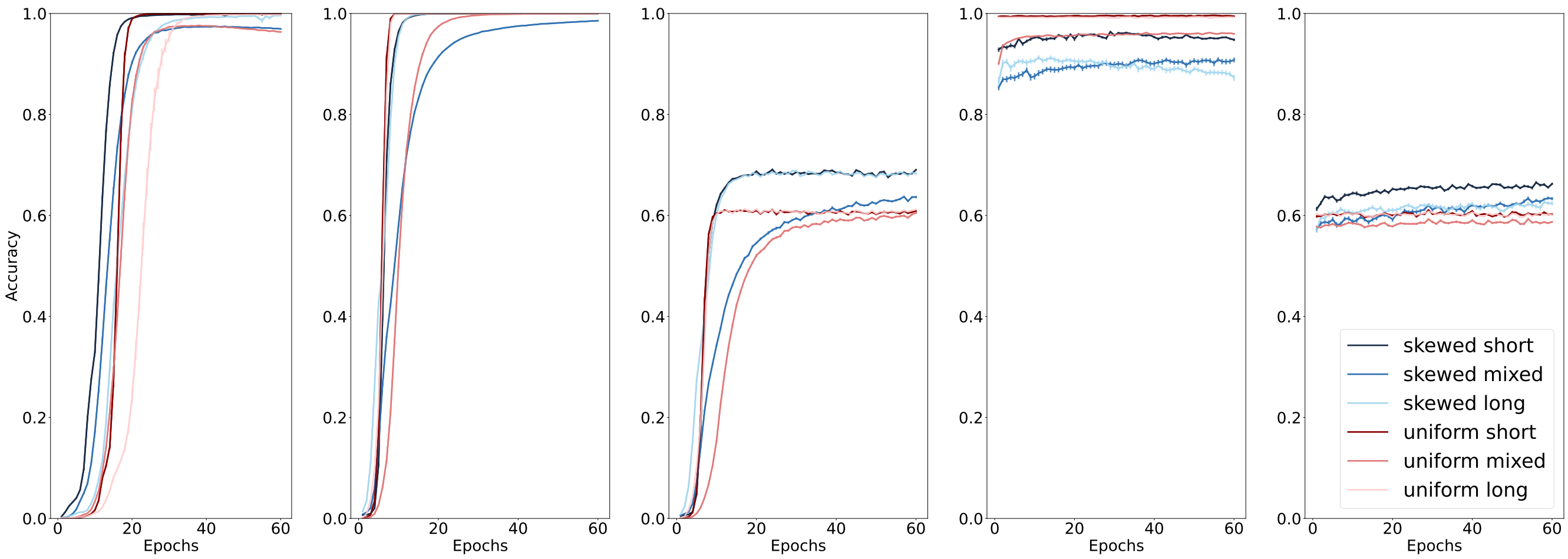

(a) Speaking (b) Listening (noise 0) (c) Listening (noise 0.1) (d) Communication (noise 0) (e) Communication (noise 0.1)

FIGURE D4 Performance of RNN-based agents in speaking, listening with noise levels 0 and 0.1, and communication with noise levels 0 and 0.1 during learning and communication in the **verb-final language** under **the *Baseline* and the noise condition**.

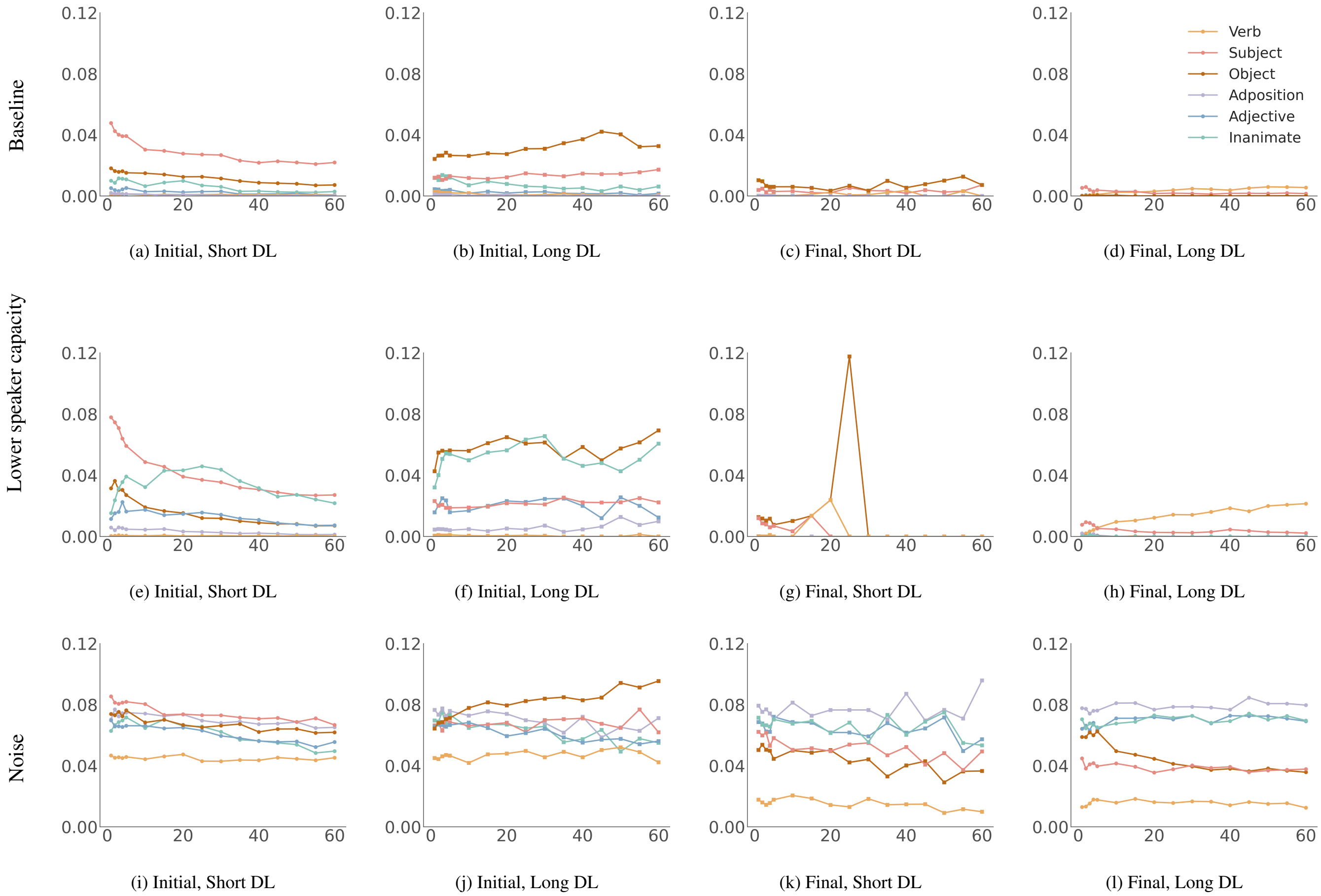

FIGURE E5 Evolution of error percentages (y-axis) for all meaning items across epochs (x-axis). Columns 1 and 2 show results for **verb-initial** languages, while columns 3 and 4 show results for **verb-final** languages. Rows correspond to different experimental conditions: *Baseline*, *Lower speaker capacity*, and *Noise*. Error rates are calculated separately for short- and long-dependency utterances. Adposition, Adjective, and Inanimate refer to adposition, adjective, and inanimate noun in the modifier phrase.
